# Structural Basis for the Functional Changes by EGFR Exon 20 Insertion Mutations

**DOI:** 10.3390/cancers13051120

**Published:** 2021-03-05

**Authors:** Mahlet Z. Tamirat, Kari J. Kurppa, Klaus Elenius, Mark S. Johnson

**Affiliations:** 1 Structural Bioinformatics Laboratory, Biochemistry, Faculty of Science and Engineering, Åbo Akademi University, 20520 Turku, Finland; mahlet.tamirat@abo.fi; 2 MediCity Research Laboratories, Institute of Biomedicine, University of Turku, 20520 Turku, Finland; kjkurp@utu.fi (K.J.K.); klaus.elenius@utu.fi (K.E.); 3 Department of Oncology, Turku University Hospital, 20521 Turku, Finland; 4 Turku Bioscience Center, University of Turku and Åbo Akademi University, 20520 Turku, Finland

**Keywords:** EGFR tyrosine kinase, exon 20 insertion mutations, non-small cell lung cancer, molecular dynamics simulation, structural biology

## Abstract

**Simple Summary:**

Non-small cell lung cancer (NSCLC) is the most common type of lung cancer that claims the lives of many worldwide. Activating mutations occurring on the epidermal growth factor receptor (EGFR) protein have been associated with the pathogenesis of NSCLC, among which exon 20 insertion mutations play a significant role. The objective of this study is to examine the dynamic structural changes occurring on the EGFR protein as a result of two common EGFR exon 20 insertion mutations, V769insASV and D770insNPG. The study further aims to uncover the mechanisms by which the insertion mutations increase kinase activity. Our results suggest that the insertion mutations stabilize structural elements key to maintaining the active EGFR conformation. Furthermore, the insertions disrupt an interaction essential in stabilizing the inactive conformation, which could drive the kinase from an inactive to an active EGFR state.

**Abstract:**

Activating somatic mutations of the epidermal growth factor receptor (EGFR) are frequently implicated in non-small cell lung cancer (NSCLC). While L858R and exon 19 deletion mutations are most prevalent, exon 20 insertions are often observed in NSCLC. Here, we investigated the structural implications of two common EGFR exon 20 insertions in NSCLC, V769insASV and D770insNPG. The active and inactive conformations of wild-type, D770insNPG and V769insASV EGFRs were probed with molecular dynamics simulations to identify local and global alterations that the mutations exert on the EGFR kinase domain, highlighting mechanisms for increased enzymatic activity. In the active conformation, the mutations increase interactions that stabilize the αC helix that is essential for EGFR activity. Moreover, the key Lys745–Glu762 salt bridge was more conserved in the insertion mutations. The mutants also preserved the state of the structurally critical aspartate–phenylalanine–glycine (DFG)-motif and regulatory spine (R-spine), which were altered in wild-type EGFR. The insertions altered the structure near the ATP-binding pocket, e.g., the P-loop, which may be a factor for the clinically observed tyrosine kinase inhibitor (TKI) insensitivity by the insertion mutants. The inactive state simulations also showed that the insertions disrupt the Ala767–Arg776 interaction that is key for maintaining the “αC-out” inactive conformation, which could consequently fuel the transition from the inactive towards the active EGFR state.

## 1. Introduction

**Figure 1:**
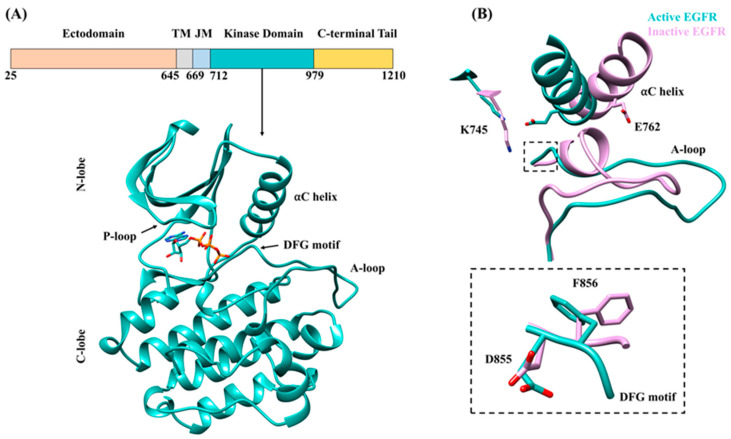
Epidermal growth factor receptor (EGFR) and structural features of the tyrosine kinase domain. (**A**) The different domains that make up the EGFR protein and structure of the intracellular kinase domain (Protein Data Bank (PDB) ID 2GS2 [10]); key structural elements and active site-bound ATP are highlighted. ATP was positioned based on AMP-PNP bound EGFR structure (PDB ID 2ITX [11]). (**B**) The active (cyan) and inactive (purple) conformations of the EGFR kinase domain as seen by comparing PDB ID 2GS2 with PDB ID 2GS7 [10]: the “αC-in” and “αC-out” conformations of the αC helix, the open and extended state of the activation loop (A-loop), the essential K745–E762 ionic interaction broken in the inactive state, and conformational differences within the aspartate–phenylalanine–glycine (DFG) motif.

Epidermal growth factor receptor (EGFR) is a membrane-bound signaling protein essential for the development of organisms, owing to its role in cell proliferation, differentiation, migration and survival [1]. EGFR belongs to the ERBB family of receptor tyrosine kinases (RTK), which additionally includes ERBB2, ERBB3 and ERBB4 [2,3]. As with other family members, the EGFR monomer is composed of an extracellular domain, a single-pass transmembrane domain (TM), an intracellular juxtamembrane (JM) segment, a cytoplasmic kinase domain and a C-terminal tail (Figure 1A) [3]. Activation of EGFR is induced by binding of growth factor to the ectodomain [3], which triggers a large conformational change from a tethered to an extended ectodomain state as seen by comparing the monomeric [4] and growth-factor bound homodimeric [5] X-ray structures. Consequently, EGFR monomers on binding a recognized growth factor, e.g., EGF, form homodimers or heterodimers with other ERBB family monomers, and the intracellular kinase domains associate as the asymmetric dimer required for activation. Activated kinase domains bind ATP and catalyze the autophosphorylation of tyrosine residues located at the C-terminal tail that act as docking sites for various other proteins, initiating intracellular signaling pathways [6,7]: in the case of EGFR homodimers, pathways such as the MAPK/ERK and PI3K-AKT that are important in cell proliferation, differentiation, migration and inhibition of apoptosis [8,9].

The EGFR kinase domain exists in an equilibrium between an inactive and active conformation (Figure 1B) and the ATP binding pocket lies between the N-terminal and C-terminal lobes (Figure 1A) [10,12,13]. Both lobes contribute structural units, such as the αC helix and activation loop (A-loop), which are critical for the regulation of EGFR kinase activity. On activation, the αC helix of the EGFR kinase domain assumes the “αC-in” conformation, where the αC helix locates near where ATP binds within the active site (Figure 1B). Consequently, a conserved ionic interaction between Glu762 of the αC helix and Lys745 of the β3 strand forms, stabilizing the active conformation. Furthermore, in the activated state, the A-loop attains an extended orientation, opening up space near the binding pocket. The aspartate–phenylalanine–glycine (DFG) motif that is located in the A-loop is placed so that the catalytic aspartate points towards the terminal phosphate group of ATP in the binding pocket. The phenylalanine of the DFG motif is embedded near the C-terminus of the αC helix with its side chain facing towards the binding site.

In contrast, in the inactive conformation of EGFR, the αC helix adopts the “αC-out” state in which the helix moves away from the binding pocket resulting in the breakage of the key Lys745–Glu762 salt bridge. Moreover, the A-loop attains a closed conformation with a small helix being formed at its N-terminus. In this inactive conformation, also known as the Src-like inactive state, the aspartate of the DFG motif maintains the same orientation as the active state. The phenylalanine—which packs against nearby hydrophobic residues in the activated conformation—in the inactive conformation adopts a different rotamer where the side chain points towards the C-terminus of the αC helix [14,15,16] (Figure 1B).

Aberrant signaling of EGFR due to overexpression or somatic mutations—amino acid replacements, deletions or insertions—has suggested a role for EGFR in many types of human cancers, the most common cancer being non-small cell lung cancer (NSCLC) [17,18,19,20]. Around 10–30% of NSCLCs involve mutations of the EGFR protein with significant ethnic variation [21]. The exon 21 L858R mutation and exon 19 deletion mutations, referred to as classical mutations, account for nearly 90% of activating EGFR mutations observed in NSCLC [22,23,24,25]. The L858R mutation is located at the A-loop of the kinase domain and is postulated to affect the integrity of a hydrophobic cluster key to maintaining the inactive EGFR conformation [10]. The exon 19 deletion mutations comprise multiple short deletions that vary in length and amino acid composition. The deletions occur at the β3-αC loop and are suggested to affect the structural stability of the adjacent αC helix by limiting its range of motions [26]. Our previous study has indeed demonstrated that the most common EGFR exon 19 deletion, Δ746ELREA750, appears to stabilize the activated kinase domain state and drives a conformational shift from the inactive state towards the active EGFR state by affecting the structural and positional stability of the αC helix [27]. NSCLC patients with the L858R and exon19 deletion mutations respond to first- and second-generation tyrosine kinase inhibitors (TKIs), such as gefitinib, erlotinib, and afatinib; however, resistance is inevitably conferred mainly due to acquisition of a second mutation, the T790M “gate-keeper” mutation [28].

**Figure 2:**
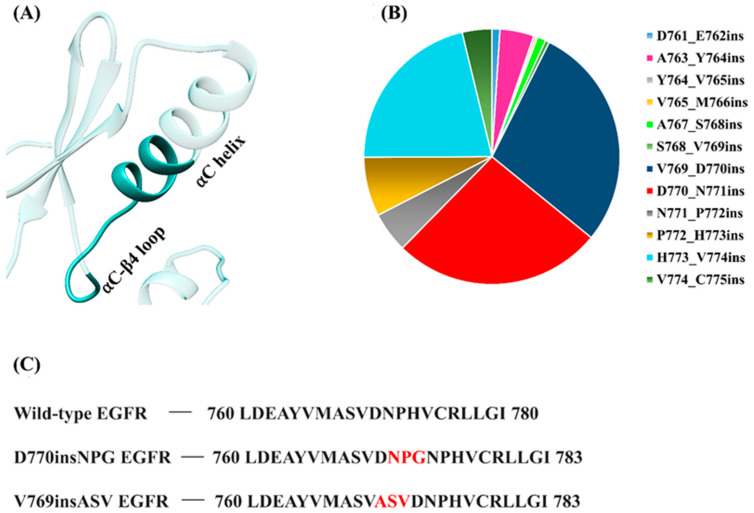
EGFR exon 20 insertion mutations. (**A**) Location of exon 20 insertion mutations (solid cyan color) within the C-terminal half of the αC helix and the αC-β4 loop of the EGFR kinase domain. (**B**) Relative frequencies of EGFR exon 20 insertion mutations observed in non-small cell lung cancer (NSCLC) as retrieved from the COSMIC database (v92) [33]. (**B**,**C**) The most commonly observed EGFR exon 20 insertion mutations in NSCLC include V769_D770ins (28.5%) and D770_N771ins (26.4%); (**C**) the insertion sequences for D770insNPG and V769insASV are highlighted in red.

Another category of activating EGFR mutations comprises the exon 20 insertion mutations. These short, in-frame insertions account for 4–10% of all EGFR mutations in NSCLC [29,30,31]. Similar to the classical activating mutations, the exon 20 insertion mutations are commonly observed in women, the Asian population and non-smokers [32]. The insertions are heterogeneous and involve the insertion of 1 to 7 amino acids, some of which result in local sequence duplication. The mutations lie between residues 762 and 774, which is within the αC-β4 loop and at the C-terminal half of the αC helix, but only a few of the insertions reside within the middle of the αC helix (Figure 2A). According to the Catalogue of Somatic Mutations in Cancer (COSMIC) database [33], the most prominent EGFR exon 20 insertion mutations occur at residues V769-D770 and D770-N771 (Figure 2B). Unlike the classical EGFR mutations, NSCLC patients with exon 20 insertions do not respond well to first- and second-generation TKIs, particularly to gefitinib and erlotinib [29]. In vitro studies and recent clinical data have shown that the approved third-generation covalent TKI, osimertinib, may have some activity towards the insertion mutations [34,35], while clinical trials are still ongoing. Another covalent TKI, poziotinib, has also shown efficacy towards EGFR exon 20 insertions in in vitro, in vivo and phase-II clinical trial studies [36]. A few exon 20 insertion mutation selective inhibitors are also currently being tested in clinical trials [37].

To date, only a single experimentally resolved structure is available for EGFR having an exon 20 insertion mutation. Yasuda et al. [38] reported the crystal structure of the human D770insNPG EGFR kinase domain that was observed to have adopted the activated kinase conformation. This study revealed key insights into the local structural changes that result from the insertion mutation and the authors hypothesized that the insertion may prevent the transition to the inactive EGFR conformation. Here, we aim to further our understanding of the structural changes induced by EGFR exon 20 insertions by probing the dynamic nature of the wild-type and mutant EGFR structures using molecular dynamics simulations (MDS). The study focuses on two insertion mutations, D770insNPG and V769insASV (Figure 2C), both of which are members of the most prevalent EGFR exon 20 insertion mutation types.

## 2. Materials and Methods

### 2.1. Structure Preparation

The three-dimensional crystal structures of EGFR kinase domains in the active form, wild-type (Protein Data Bank (PDB) ID 2GS2, 2.8 Å resolution; chain A [10]) and D770insNPG (PDB ID 4LRM, 3.5 Å resolution; chain B [38]), were obtained from the Protein Data Bank (PDB [39]) and visualized using Chimera [40] and Bodil [41]. The bound ligand in 4LRM was removed and missing structural elements in both EGFR structures were built by accessing these segments from other EGFR structures (PDB IDs 1M14, 2.6 Å resolution; chain A [42], 3W2S, 1.9Å resolution; chain A [43]). The active conformation structure of the V769insASV EGFR was modeled with Modeller [44] using the wild-type active EGFR structure as a template. The inactive state wild-type EGFR structure (PDB ID 2GS7, 2.6 Å resolution; chain A [10]) was retrieved, which also served as a template to model the inactive state of the D770insNPG and V769insASV EGFR structures. The residue numbering in the PDB files (Supplementary Files S1–S18) differ from each other: Val769 in the numbering scheme used throughout this manuscript corresponds to Val745 in 2GS2, Val769 in 4LRM, and Val745 in 2GS7. The above six structures were prepared with the protein preparation wizard in Maestro [45] by incorporating hydrogen atoms, optimizing hydrogen bonds, determining protonation states of ionizable side chains at pH 7.0 and performing restrained energy minimization.

### 2.2. Molecular Dynamics Simulations

All-atom molecular dynamics simulation (MDS) was carried out on the prepared wild-type and insertion mutant EGFR structures with the AMBER program (Version 18, University of California, San Francisco, CA, USA) [46], utilizing the ff14SB [47] force field. The proteins were solvated in an octahedral box with the TIP3P water model [48] ensuring a distance of 10 Å between the protein surface atoms and the box periphery. Na^+^ ions were added to the solvated box to neutralize the systems and extra Na^+^/Cl^−^ ions were incorporated to attain a salt concentration of 0.15 M. The systems were then processed by a four-stage simulation protocol, which is discussed in detail here [49]. Initially, 5000 cycles of energy minimization were performed using the steepest descent and conjugate gradient methods. The solute atoms were restrained with a 25 kcal mol^−1^ Å^−2^ force, which was lowered gradually to finalize with an unrestrained minimization. The systems were subsequently heated to 300 K with a solute-atom restraint force of 10 kcal mol^−1^ Å^−2^. Afterwards, equilibration was carried out for 900 ps by systematically reducing the solute atom restraint force to 0.1 kcal mol^−1^ Å^−2^. A 5 ns unrestrained simulation was carried out to complete the equilibration stage. Finally, a 600 ns production simulation was conducted at constant pressure (1 bar) and temperature (300 K), and coordinates were saved every 10 ps. Periodic boundary conditions were ensured, the particle-mesh Ewald method [50] was used for long-range electrostatic interactions and a 9 Å distance cut-off was assigned for non-bonded interactions. The simulations were carried out in duplicate by varying initial velocities in order to broadly sample the conformational space. In total, 7.2 μs long simulations were carried out for this study, which were performed on V100 GPUs provided by the CSC IT Center for Science.

### 2.3. Analysis

The trajectories of the simulations were analyzed using the programs CPPTRAJ [51] and VMD [52]. The stability of the proteins was examined by computing the backbone-atom root-mean-square deviations (RMSD), Cα-atom root-mean-square fluctuations (RMSF) and Cα-atom radii of gyration (Rgyr) from the trajectories. Hydrogen bond interactions were required to have a bond distance ≤3.5 Å and a bond angle ≥135°. Secondary structure analysis was carried out using the DSSP method [53] implemented in CPPTRAJ. The average correlations between the motions of the kinase domain residues were computed based on their Cα-atoms using CPPTRAJ.

In order to determine the dominant motions of the wild-type and mutant EGFRs during the MDS, principal component analysis (PCA) was carried out. For PCA, a covariance matrix of atomic coordinates is produced, which in this study was based on the Cα atoms of the proteins. Diagonalization of the generated covariance matrix results in eigenvectors that are defined by eigenvalues, which, respectively, describe the direction and amplitude of motions. The CPPRAJ program was employed to perform PCA and the resulting data were analyzed with the Normal Mode Wizard [54] available in VMD.

## 3. Results

Amino-acid replacements, deletions and insertions have been identified within the EGFR family that inactivate and activate kinase function, linking kinases to various diseases [55] resulting from changes to signaling affecting cell growth and differentiation [56,57,58]. Because of the inherent dynamic nature of the EGFR receptor as a whole—and especially for the kinase domain whose enzymology triggers signaling events—it is necessary to explore the dynamic consequences induced by function-altering mutations in order to understand the process at the molecular level.

In this study, we set out to investigate the dynamic structural alterations on the EGFR kinase structure resulting from two activating exon 20 insertion mutations observed in NSCLC; D770insNPG and V769insASV. The wild-type and insertion mutant EGFR kinase domains were simulated to explore changes on local structure, global dynamics and interactions. The effects of the mutations on both the active and inactive EGFR conformations were probed. For each EGFR, two independent simulations were carried out for 600 ns to enhance sampling of the conformational space. Below, we mainly discuss data from simulation 1, since the results are largely consistent with those observed in simulation 2. However, any substantial differences between the two simulations are discussed.

### 3.1. Structural Overview and Dynamics of Mutant and Wild-Type EGFR Kinase Domains

**Figure 3:**
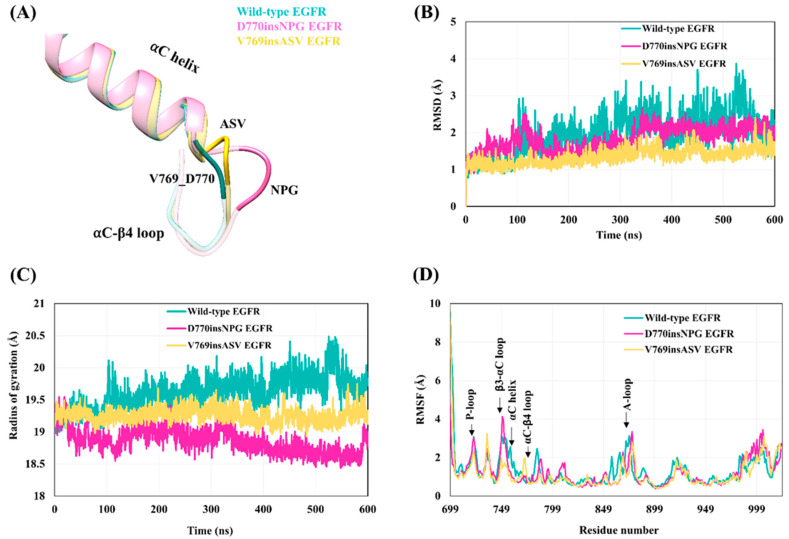
Stability of the wild-type and insertion mutant EGFR kinase domains during the 600 ns molecular dynamics simulation (MDS). (**A**) Starting structure of the wild-type EGFR superposed on the structure of the D770insNPG exon 20 insertion mutant and model structure of the V769insASV exon 20 insertion mutation. The mutations place the additional residues at the C-terminus of the αC helix. (**B**–**D**) Parameters describing the overall structural variation—i.e., stability—for the wild-type, D770insNPG and V769insASV EGFR kinase domains during MDS: (**B**) backbone-atom root-mean-square deviations (RMSD); (**C**) Cα-atom radii of gyration (Rgyr); and (**D**) Cα-atom root-mean-square fluctuations (RMSF).

The crystal structure of the active state D770insNPG EGFR kinase domain (PDB ID-4LRM [38]) shows that the insertion of the three residues (Figure 2C) at the end of the αC-helix and the beginning of the αC-β4 loop results in the formation of a β-turn, stabilized by a hydrogen bond between the main-chain oxygen atom of D770 and the 2.3 Å distant main-chain nitrogen atom of *Gly773* (italics indicate an inserted residue; the numbering of the remainder of the sequences of the mutant EGFRs is offset by three in comparison to wild-type EGFR), extending the length of the αC-β4 loop region (Figure 3A). With the V769insASV mutation or A767-V769 duplicate, in which the three C-terminal residues of the αC helix ASV are duplicated, i.e., MASVD → MASV*ASV*D (Figure 2C), the structural model shows that the three inserted residues, *ASV*, form an additional helix-turn at the C-terminus of the αC helix (Figure 3A) and the main-chain nitrogen of the inserted valine *V772* is within hydrogen bonding distance (2.9 Å) of the main-chain oxygen of Val769.

The overall structural dynamics of the active state wild-type and insertion-mutant EGFR kinase domains were probed with MDS. Backbone-atom RMSD calculations revealed that the kinase domain of the insertion mutants is more stable with smaller and less variable deviation values (D770insNPG: 1.7 ± 0.3 Å; V769insASV: 1.34 ± 0.2 Å), than the wild-type EGFR (1.95 ± 0.5 Å), and the wild-type EGFR structure showed higher deviations as the simulation progressed (Figure 3B). The compactness of the three EGFRs was assessed by computing the Cα atom Rgyr, which represents the distribution of atoms from the molecular center of mass (Figure 3C). The mutant EGFRs exhibited a more compact packing of the kinase core in comparison to wild-type EGFR as reflected in the mutants’ smaller average values of Rgyr, with the Rgyr of D770insNPG (18.86 ± 0.25 Å) lowest overall and decreasing during the simulation, followed by V769insASV (19.3 ± 0.32 Å); and the higher value of Rgyr (19.7 ± 0.41 Å) observed for wild-type EGFR increased during the progression of the simulation.

The flexibility of the main chain at each residue was assessed by computing the Cα-atom RMSF of the wild-type and mutant EGFR kinase domains (Figure 3D). The results show that the wild-type and mutant proteins generally display similar stability profiles, with the αC helix, P-loop, β2-β3 loop, β3-αC loop and A-loop of the kinase domain showing relatively higher fluctuations than the rest of the structural elements. At the N-terminus of the αC-β4 loop that accommodates the three amino-acid insertion in both mutant EGFRs, the D770insNPG EGFR exhibited comparable flexibility to that of wild-type EGFR. The additional helix turn in the V769insASV mutant, stabilized by a hydrogen bond, shows some flexibility: the RMSF over the inserted *Ser771* and *Val772*, respectively, varies between 1.0 Å (simulation 2) and 1.8 Å (simulation 1). A distinct difference between the wild-type and mutant EGFRs is the flexibility of residues within the αC helix that precede the site of the mutation. The αC helix was less flexible for the insertion mutants as compared to wild-type EGFR. 

### 3.2. Impact of the Exon 20 Insertions on the αC Helix of the Active State of EGFR

**Figure 4:**
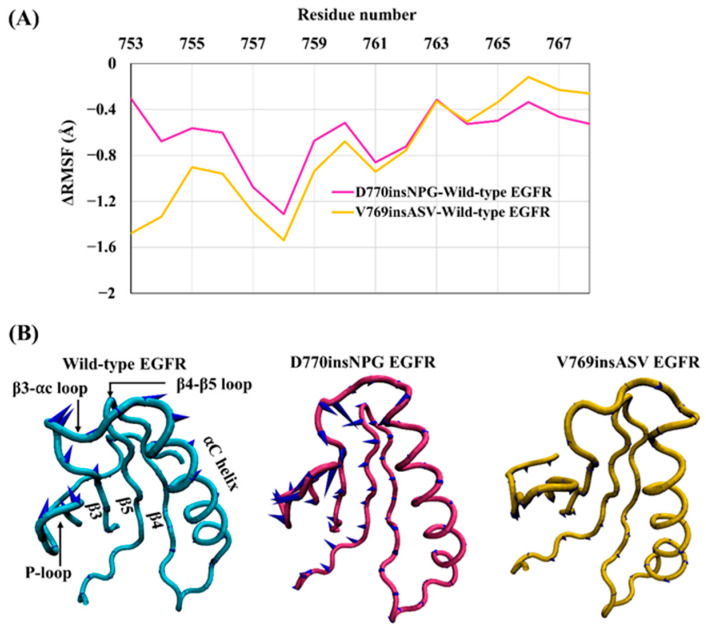
Dynamics of the αC helix. (**A**) RMSF difference between the αC helices of the wild-type EGFR kinase domain and those of the mutants D770insNPG, and V769insASV. The N-terminal half of the αC helices of D770insNPG and V769insASV show greater RMSF differences from wild-type EGFR. (**B**) Principal component analysis (PCA) of the wild-type and insertion mutant EGFRs, with porcupine plots depicting the motions of the αC helix and the N-lobe structural units in the first principal component. The “cones” of the wild-type αC-helix are larger in size (reflecting a larger magnitude of the movement) and are directed outwards, whereas the mutants exhibit smaller cones that face inwards, indicating a more stable αC helix and the direction of movement supports the EGFR activated state. Similarly, the majority of the N-lobe of the insertion mutant structures show an inward movement while wild-type EGFR largely exhibits an outward motion.

The conformation and stability of the αC helix is key in regulating the switch between active and inactive EGFR kinase states. During the simulations, the αC helix of wild-type EGFR was more flexible in comparison to the D770insNPG and V769insASV EGFRs—especially at the N-terminal half of the αC helix—as shown by the negative values for the difference in the RMSF overall residues comprising the αC helix (Figure 4A). This observation is likely linked to the increased interactions present at the C-terminus of the mutant αC helices: in both instances, interactions are formed between the inserted residues and the αC helix, αC-β4 loop and αE helix. These interactions would contribute to further stabilizing and holding the αC helix in place.

In order to assess the dominant modes of motions recorded for the αC helix during the MDS, PCA was applied to the trajectories from MDS. Motions described by the first principal component show that the αC helix of the mutant EGFRs is more stable than that of the wild type as represented by the smaller size of the cones of the porcupine plot (Figure 4B). PCA also revealed that while the mutants D770insNPG and V769insASV demonstrate an inward movement of the αC helix (towards the binding pocket), the wild-type structure supports an outwards motion of the αC helix, a move required in transitioning from a catalytically active EGFR state towards the inactive state. These results suggest that the insertion mutants would alter EGFR kinase function by helping to preserve the active conformation of EGFR. The PCA analysis additionally suggests that these are concerted motions that affect the entire catalytic region: the direction of motion of the αC helix is correlated to the motions of most of the N-lobe structural elements, such as the P-loop, β3-αC loop, β4-β5 loop, and the β3 and β4 strands. These structural units of wild-type EGFR move outwards from the catalytic and ATP binding pocket as does the αC helix. In contrast, in the D770insNPG and V769insASV mutant forms, the movement is largely inwards, similar to the movement of the αC helix. In line with this observation, a cross-correlation analysis between residues of the kinase domain of the wild-type and mutant EGFRs also revealed a strongly correlated motion among the structural units of the N-lobe of the kinase domain, which includes the αC helix (Figure S1).

**Figure 5:**
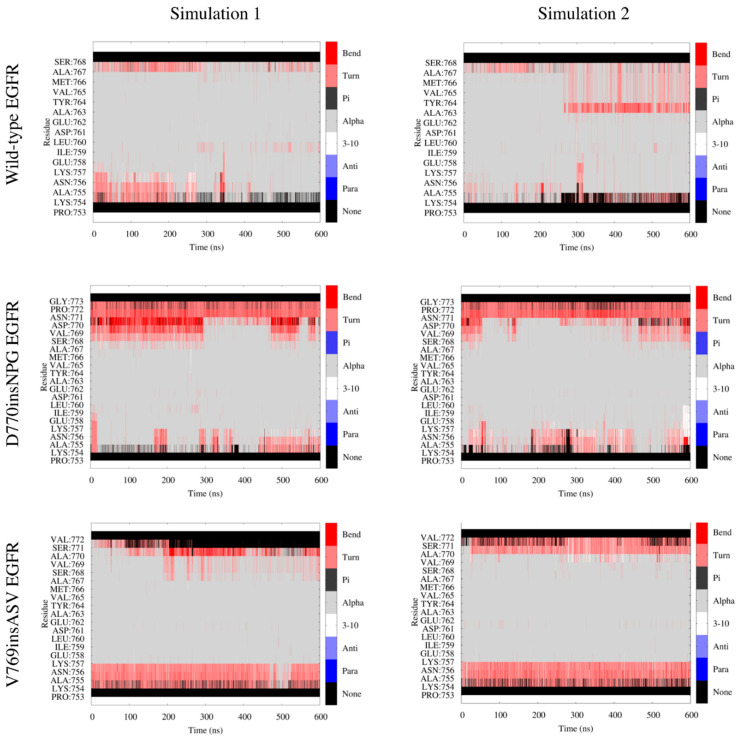
Secondary structure representation of individual residues along the αC helix (inserted residues also shown) during the duplicate simulations of the wild-type (residues 753–768), D770insNPG (753–773) and V769insASV (753–772) EGFRs. The helical structure of the central and C-terminal end of the αC helix was more conserved in the insertion mutants, whereas wild-type EGFR displayed turns and bends during the simulations. Residues at the N-terminal end of the αC helix largely attained turns in all three EGFRs.

Analysis of the per-residue secondary structure of the αC helix during MDS can reveal localized fluctuations away from an ideal helix. With the insertion mutants, the central region and C-terminal end of the αC helix are conformationally more conserved in contrast to wild-type EGFR, which diverges from an ideal helix in multiple frames (i.e., turn and bend designations in Figure 5). The inserted residues in D770insNPG EGFR forms turns during the simulation, whereas in V769insASV both turn and helix conformations were formed. At the N-terminal end of the αC helix, both the wild-type and D770insNPG mutant EGFR had comparable secondary structure profiles, with several frames having turns and bends, but these were more pronounced in the V769insASV EGFR simulation. The structural integrity of the N-terminus of the αC helix is more compromised in the V769insASV EGFR likely due to the interactions this region is making with the adjacent β3-αC loop. Sampled conformations reveal a close hydrophobic interaction between Ala755 of the αC helix and Ala750 of the β3-αC loop that changes the helical structure at the N-terminus (Figure S2). The same interaction is also likely the reason for the better stability of the β3-αC loop in the V769insASV EGFR as compared to D770insNPG EGFR (Figure 3D). D770insNPG EGFR, which has a better helical integrity at the N-terminus of the αC helix, lacks the close interaction between Ala755 and Ala750 (Figure S2). As a result, the β3-αC loop attains different conformations, accounting for the higher RMSF of this region in D770insNPG EGFR in contrast to V769insASV EGFR. Indeed, the β3-αC loop is the location for multiple activating deletion mutations, where the shortening of this loop is postulated to impart structural stability to the nearby αC helix [27]. 

Taken together, the simulations reveal that the EGFR insertion mutations, D770insNPG and V769insASV, impart structural and positional stability along the αC helix. This is likely due to the hydrogen-bond stabilized secondary structures in the form of a β-turn in D770insNPG and an additional turn of the αC helix in V769insASV, both located at the C-terminus of the αC helix. These interactions would serve as an anchor to restrain the helix in its active state conformation.

### 3.3. Interactions Due to the Insertion of Residues in the EGFR Exon 20 Mutants

**Figure 6:**
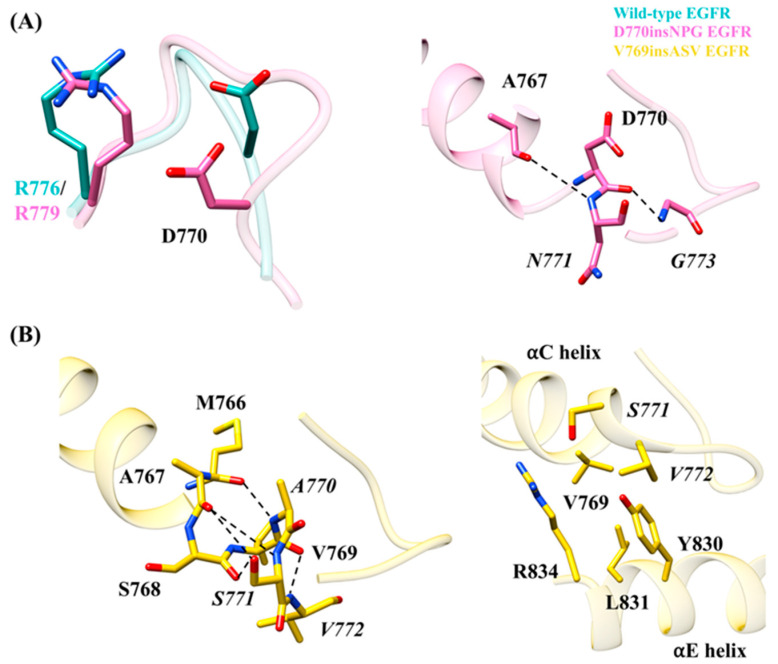
Interactions observed at the mutation sites and indicated on the starting EGFR structures. These interactions were observed during MDS. A) D770insNPG EGFR and B) V769insASV EGFR. (**A**) Placement of Arg776 (wild type)/Arg779 (mutant) and Asp770 (left) and comparison with wild-type EGFR. In D770insNPG EGFR, due to the insertions, Asp770 is positioned slightly closer to Arg779 of the αC-β4 loop. Interactions (right, dotted lines) crucial for stabilizing the β-turn formed by the inserted residues in D770insNPG EGFR. (**B**) Hydrogen bonding interactions (left, dotted lines) observed maintaining the additional helix turn formed by the V769insASV insertion at the C-terminus of the αC helix. Amino acids (right) that are integral for the interaction between the C-terminus of the αC helix and the αE helix in V769insASV EGFR.

Insertion of the three residues in the EGFR mutants D770insNPG and V769insASV results in a structural change at the end of the αC helix and alters the interactions taking place in their vicinity. In D770insNPG EGFR, the insertion leads to a change in the location of Asp770, bringing the side chain slightly closer to the C-terminus of the αC-β4 loop (Figure 6A) in comparison to wild-type EGFR (Figure 6A). In D770insNPG, a hydrogen bond interaction between Asp770 and Arg779 (Arg776 in wild-type EGFR) of the αC-β4 loop, connecting the C-terminus of the αC helix and the αC-β4 loop, is observed in 83% of the trajectory, whereas this is the case in only 25% of the trajectory from the wild-type simulation. Additionally, during the D770insNPG EGFR simulation, two hydrogen bonds were observed with the inserted residues, both helping to stabilize the newly formed β-turn (Figure 6A): the main-chain oxygen atom of Asp770 hydrogen bonded with the nitrogen atom of *Gly773* (49%), and the main-chain nitrogen atom of the inserted *Asn771* hydrogen bonded with the main-chain oxygen atom of Ala767 (10%). In wild-type EGFR, the lack of restraining interactions linked to the inserted residues, together with fewer recorded interactions between Asp770 and Arg776, predisposes the C-terminus of the αC helix to more flexibility. With the V679insASV EGFR mutant, the three inserted residues frequently form an additional helix-turn at the C-terminus of the αC helix, which was maintained by a series of hydrogen bonds (Figure 6B) that includes *Ala770* N–Met766 O (86%), *Ser771* OH–Ser768 O (11%), *Ser771* OH–Ala767 O (16%), *Ser771* N–Ala767 O (16%) and *Val772* N–Val769 O (9%). Furthermore, a hydrophobic interaction between the side chains of *Val772* and Tyr830 and a hydrogen bond between side chains of *Ser771* and Arg834 reinforces the contact between the αC and αE helices, further stabilizing the C-terminus of the αC helix (Figure 6B). In the simulations of both the D770insNPG and V769insASV mutant EGFRs, Val769 of the αC helix is placed in closer proximity to Leu831 of the αE helix, respectively, with an average Cα-atom distance of 8.0 ± 0.5 Å and 7.5 ± 0.4 Å, as compared to 9.5 ± 0.7Å for wild-type EGFR (Figure S3). Consequently, a stronger interaction between Val769 and Leu831 may take place in the mutant forms, which would also help stabilize the active state conformation of EGFR. Taken together, the simulations reveal that the insertion mutations provide additional interactions that could impart additional stability at the αC helix. 

### 3.4. Effect of Exon 20 Insertions on the Lys745–Glu762 Salt Bridge of the EGFR Active State

**Figure 7:**
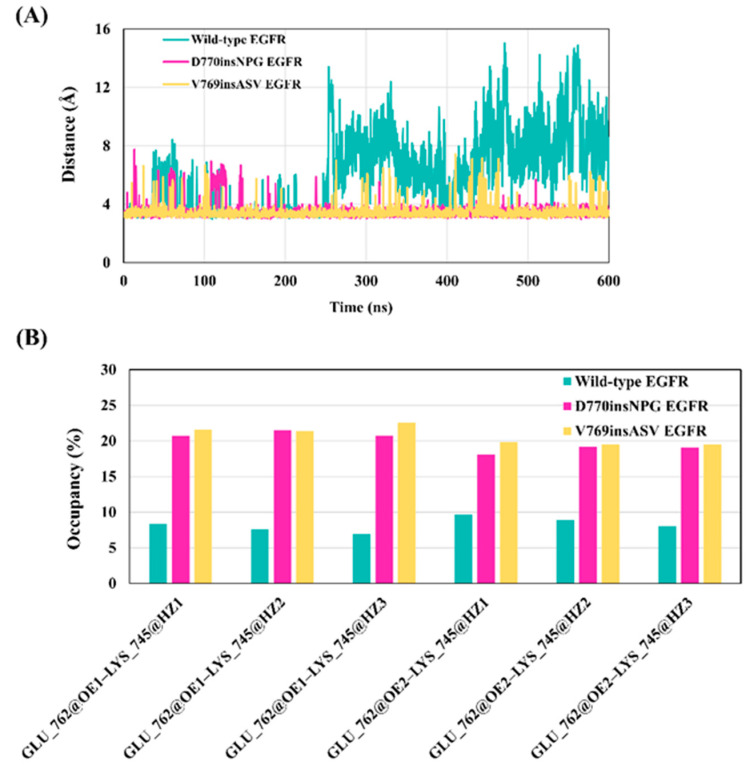
Distance and hydrogen bond occupancy between Lys745 and Glu762 during the wild-type and insertion mutants MDS. (**A**) Distance between the Nζ atoms of Lys745 and Cδ atom of Glu762 during the wild-type and insertion mutant simulations. The mutants preserved a shorter and less variable distance as compared to wild-type EGFR that exhibits a larger distance that implies that the salt bridge is broken. (**B**) The frequency of hydrogen bonds between the amine and carboxyl side-chain oxygen atoms of Lys745 and Glu762. The bonds were more frequently observed during the insertion mutant simulations than for the wild-type EGFR.

A salt bridge between a glutamate from the αC helix and a lysine from the β3 strand is critical for EGFR kinase activity (Figure 1B) [15]. In the “αC-in” active kinase conformation this salt bridge optimally positions the lysine to interact with the α- and β-phosphates of ATP, aiding in the catalytic process; in the “αC-out” inactive conformation, the Lys745–Glu762 salt bridge is broken. To assess the effect of the insertion mutations on the state of the Lys745–Glu762 salt bridge, the distance between the Cδ atom of Glu762 and the Nζ atom of Lys745 (to attain a single central measurement, the Cδ side-chain atom of Glu762 was used as opposed to the OE1/OE2 atoms) were monitored for the wild-type, D770insNPG and V769insASV EGFRs during MDS. The average distance between Lys745 and Glu762 was shorter and more consistent for the mutant EGFRs (3.5 ± 0.5 Å for D770insNPG and 3.45 ± 0.46 Å for V769insASV; Figure 7A); in contrast, wild-type EGFR exhibited a longer average distance between the two residues (6.0 ± 2.6 Å), particularly from 250 ns onwards during the simulation, indicating that the salt bridge had broken. Further supporting this observation is the hydrogen bond occupancy between the side-chain polar atoms of Glu762 (OE1, OE2) and Lys745 (NH3): the percentage occupancy in the mutants was about twice that seen for wild-type EGFR. (Figure 7B). These findings suggest that the key Lys745–Glu762 salt bridge is firmly maintained in the mutant EGFRs unlike the wild-type, likely due to the better positional and structural stability of the αC helix observed for the exon 20 insertion mutants.

### 3.5. Effect of the Insertion Mutations on the Active State DFG Motif and Regulatory Spine

**Figure 8:**
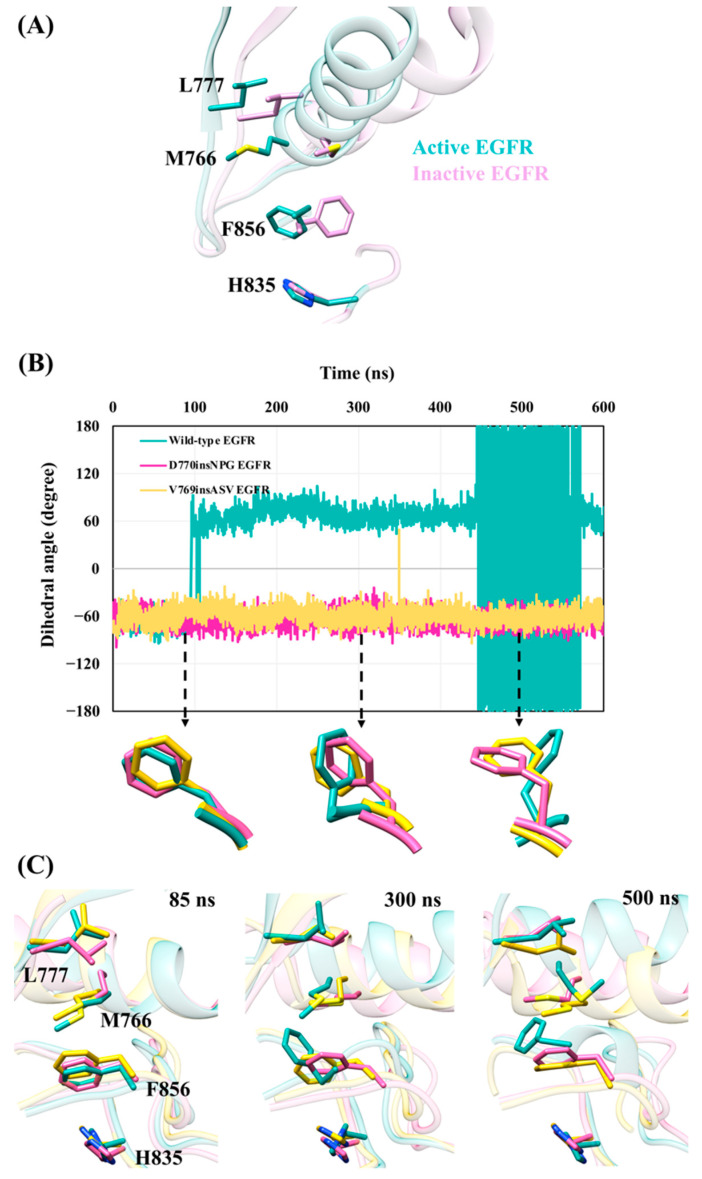
The insertion mutants appear to maintain the orientation of phenylalanine (Phe856) of the DFG motif and stabilize the R-spine of the EGFR active state. (**A**) Superimposed active and inactive conformations of the EGFR kinase with residues of the R-spine highlighted. (**B**) The χ1 dihedral angle of Phe856 of the DFG motif during the simulations of wild-type, D770insNPG and V769insASV EGFRs. The χ1 angles of the Phe856 side chain during the simulations of the D770insNPG and V769insASV EGFR mutants were consistent with the EGFR active state. In contrast, the χ1 dihedral angle of Phe856 of the wild-type EGFR deviated towards the inactive state’s conformation starting from 100 ns onwards. It is noteworthy that during 450 to 575 ns the χ1 angle exceeded 180° in multiple frames, which is represented by negative values due to the χ1 dihedral angle range limit (180° to −180°). Therefore, an angle of 181° is represented as −179°, creating a seemingly significant variation on the graph, although in reality the angle difference is only 1°. (**C**) Sampled conformations at 85,300 and 500 ns of the wild-type and insertion mutant EGFRs, highlighting the positioning of the R-spine residues. At 500 ns the orientations of both Phe856 and Met766 change in the wild-type EGFR, disrupting the R-spine assembly.

The DFG motif of the kinase domain plays a crucial role in the regulation of kinase activity [13,14,15]. In the active state of the EGFR kinase, aspartate of the DFG motif is oriented towards the ATP binding site (Figure 1B) and coordinates with a magnesium ion, fulfilling an essential role in the catalytic activity of the enzyme. The phenylalanine on the other hand is embedded in a pocket near the αC helix forming part of the regulatory spine (R-spine). The R-spine, which is also crucial for the structural stability of the kinase domain, is composed of four non-consecutive hydrophobic residue side chains that pack against their spatial neighbor(s) when they are aligned in the active conformation of EGFR [3]. In the inactive kinase state, the disposition of the R-spine residues is disrupted, mainly due to the “αC-out” conformation of the αC helix. Consequently, phenylalanine of the DFG motif attains an orientation different from the active state (Figure 8A).

In order to examine the effect of the D770insNPG and V769insASV insertion mutations on the EGFR DFG motif, the orientation of phenylalanine (Phe856) during the simulations was monitored by measuring the χ1 side-chain dihedral angle (Figure 8B). The average χ1 angle for Phe856 in the simulations of both D770insNPG EGFR (−65.2 ± 9.9°) and V769insASV EGFR (−59.2 ± 10.25°) was similar to that (χ1 = −63.8°) observed in the wild-type crystal structure of the EGFR active conformation. The average χ1 dihedral angle observed for the simulated wild-type EGFR varied between 68.1 ± 10° and 178.7 ± 10.4° (simulation 1) and −54.7 ± 12° (simulation 2). During 100 to 450 ns of simulation 1, the χ1 angle (68.1 ± 10°) is in a similar range as Phe856 observed in the crystal structures of inactive EGFRs (PDB IDs 3POZ [59] and 3W2S [43]) that have a χ1 angle in the range 50°–55°. Whereas between 450 and 575 ns of simulation 1, the χ1 angle of Phe856 averaged 178.7 ± 10.4°, which highly resembles the Phe856 rotamer observed in other inactive EGFR structures (PDB IDs 2GS7 [10], 4HJO [60]) with χ1 in the range 163°–169°. In the latter orientation, Phe856 has left the pocket near the C-terminus of the αC helix and the R-spine arrangement dissolves.

Among the four non-consecutive residues that make up the R-spine, Met766 is located at the C-terminus of the αC helix. In the active EGFR kinase structure, Met766 is aligned parallel to the rest of the residues of the R-spine (Figure 8A). While this relative arrangement was conserved during the simulations of the D770insNPG and V669insASV mutant EGFRs, the assembly was disrupted in simulation 1 of wild-type EGFR, both from the movement of Met766 away from the R-spine due to the movement of the αC helix together with the reorientation of the Phe856 side chain (Figure 8C). These observations collectively suggest that the structural stability and positional constraint on the αC helix, particularly at its C-terminus, imparted by the D770insNPG and V769insASV insertion mutations, strengthens the integrity of the DFG motif and the R-spine by maintaining the orientation of both Phe856 and Met766 and their respective hydrophobic interactions with Leu777 and His835. As a result, the insertion mutants would strongly support maintenance of the active conformation of EGFR. In contrast, in wild-type EGFR, which lacks the same degree of structural and positional stability of the αC helix, Phe856 and Met766 were observed to attain orientations that alter the conformation of the DFG motif and break the R-spine assembly and would likely promote a transition from the active conformation towards the inactive conformation.

### 3.6. Structural Changes and Interactions at the Mutation Sites in Inactive D770insNPG and V769insASV EGFRs

**Figure 9:**
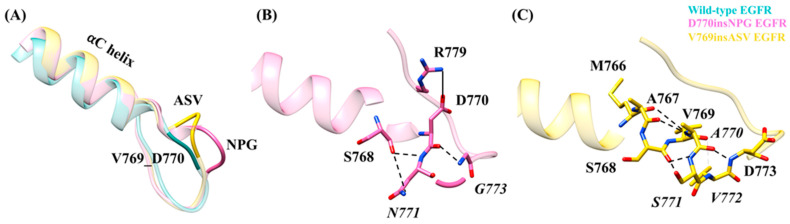
Inactive EGFRs: Structural changes and interactions in D770insNPG and V769insASV at the site of the insertion mutations. (**A**) Superimposed starting structures of wild-type, D770insNPG and V769insASV inactive EGFRs, highlighting the location of the insertions. Interactions involving the inserted residues in the inactive (**B**) D770insNPG and (**C**) V769insASV EGFRs. The frequently formed D770–R779 interaction in D770insNPG EGFR is also shown.

The insertion of the three residues in the inactive state D770insNPG and V769insASV EGFRs resulted in local structural changes similar to the ones observed in the active conformations of the mutant EGFRs. The insertions in the inactive D770insNPG EGFR model result in the formation of a β-turn at the beginning of the αC-β4 loop, whereas in the V769insASV EGFR structure the inserted residues extend the helical-turn at the end of the αC helix (Figure 9A). During the inactive D770insEGFR simulation multiple interactions including Ser768 O–*Asn771* N (61%), Asp770 O–*Gly773* N (50%) and Ser768 O–*Asn771* ND2 (26%) were formed involving the inserted residues (Figure 9B). Furthermore, in D770insNPG EGFR due to the close positioning of Asp770 and Arg779 of the αC-β4 loop, a hydrogen bond between the two residues occurred during 88% of the D770insNPG EGFR simulation, which was less frequently recorded in the wild-type EGFR simulation (26%). During the inactive V769insASV EGFR simulation several hydrogen bonds including Ser768 O–*Ser771* N (64%), Ala767 O–*Ala770* N (63%), *Ala770* O–Asp773 N (44%), Ser768 O–*Ser771* OH (42%), *Val772* N–Val769 O (31%) and Met766 O–*Ala770* N (17%) helped maintain the additional helix-turn formed at the C-terminus of the αC helix (Figure 9C).

### 3.7. Inactive Wild-Type and Mutant Simulations: The State of Key Structural Elements of the Kinase Domain

**Figure 10:**
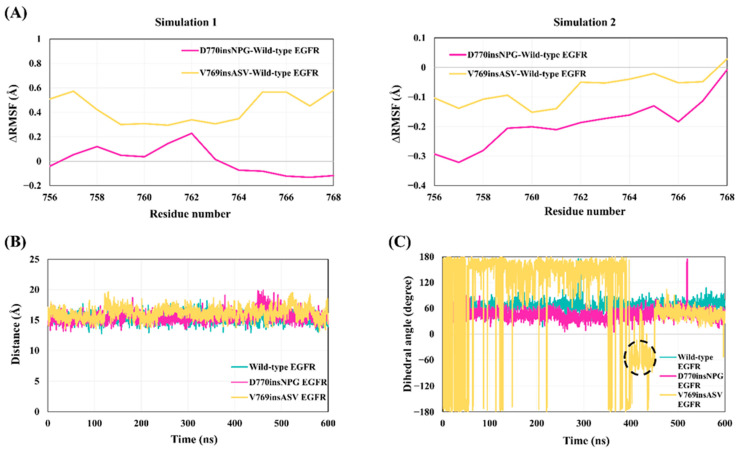
Stability of the αC helix, the broken-state of the Lys745–Glu762 salt bridge and conformation of Phe856 of the DFG motif in the wild-type and mutant inactive EGFRs. (**A**) RMSF difference between residues of the αC helix from the wild-type and insertion mutant EGFRs in simulation 1 (left) and 2 (right). (**B**) Distance between the side-chain atom Nζ of Lys745 and Cδ of Glu762; the salt bridge did not form during the simulations. (**C**) The χ1 torsion angle of Phe856 of the DFG motif. The wild-type and D770insNPG EGFRs strictly maintain the inactive state conformation of Phe856 throughout the 600 ns simulation, whereas the V769insASV EGFR visits the active state conformations between 400 and 450 ns of the simulation (encircled).

The structural stability of the αC helix of the wild-type and mutant inactive EGFRs was assessed with Cα-atom RMSF calculation for the residues of the helix (Figure 10A), which are three amino acids shorter at the N-terminal end than in active-state EGFR. The analysis of simulation 1 revealed that residues of the αC helix in the mutant EGFRs are slightly more flexible than the wild type, in particular for the V769insASV EGFR. Simulation 2, however, showed that the mutants are marginally less flexible. Similar secondary structure analysis profiles, however, were observed for the αC helix of the wild-type and insertion mutant EGFRs: the helical structure of the central part of the αC helix is conserved, whereas the N-termini exhibited turns in multiple conformations (Figure S4). In the mutant EGFRs, the inserted residues at the C-terminal end of the αC helix mainly formed turns and bends, which are frequently present in D770insNPG EGFR. The V769insASV EGFR, in contrast, exhibited a 3_−10_ helix for this region, suggesting an extension of the helical conformation at the C-terminus of the αC helix.

Superimposed conformations from the inactive simulations of the wild-type and insertion mutant EGFRs show that the inactive conformation of the kinase domain was maintained in all three EGFRs, with the αC helix adopting the “αC-out” position and the A-loop helix pressing against the αC helix (Figure S5). Furthermore, the Lys745–Glu762 salt bridge was broken in both the wild-type and mutant EGFRs (Figure 10B), with an average distance of 15 Å between the Nζ and Cδ side-chain atoms of Lys745 and Glu762. Lys745, instead, made ionic interactions with Asp855 of the DFG motif, which is formed for 83%, 72% and 90% of the simulation time of the wild-type, D770insNPG and V769insASV EGFRs, respectively; Glu762 formed ionic interactions with Lys860 of the A-loop, which was similarly maintained in the wild-type (71% of the simulation time) and mutant EGFRs (D770insNPG 71%; V769insASV 75%). Analysis of the DFG motif revealed the maintenance of the inactive state orientation of Phe856 in both the wild-type and D770insNPG EGFRs (Figure 10C). However, during 400 to 450 ns of simulation 1 of V769insASV EGFR, Phe856 attained a χ1 dihedral angle similar to the active state EGFR, which might indicate that the V769insASV mutation could promote structural changes characteristic of a shift from the inactive towards the active state EGFR conformation. This notion, however, would be strongly supported by longer simulations, as a transition between EGFR active–inactive states is captured in simulations ranging on the microsecond scale [61], which are not fulfilled by our 600 ns simulations.

### 3.8. Impact of the Exon 20 Insertion Mutations on the Ala767–Arg776 Interaction in the EGFR Inactive Conformation

**Figure 11:**
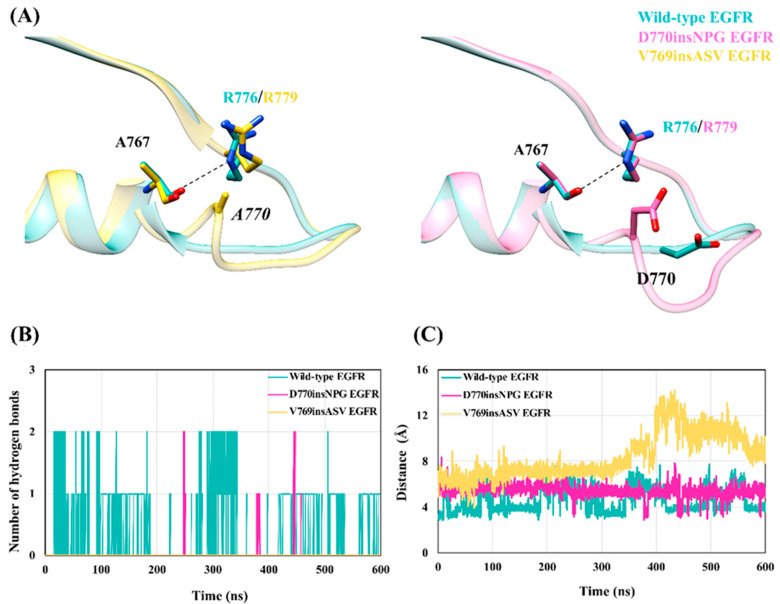
The EGFR D770insNPG and V769insASV insertion mutations may block interaction between Ala767 and Arg776 (wild type)/Arg779 (mutant) that helps maintain the inactive state. (**A**) Superposed starting structures (left) of wild-type and V769insASV EGFRs and disposition of Ala767 and Arg776/Arg779, and the inserted *Ala770*. *Ala770* wedges between Ala767 and Arg779, preventing the formation of the hydrogen bond between the two residues. Superimposed starting structures (right) of the wild-type and D770insNPG inactive EGFRs: Asp770 in the insertion mutant is oriented close to Arg779 in comparison to wild-type EGFR. Hence, Asp770 would interfere with the optimal placement of Arg779 to interact with Ala767. (**B**) The number of hydrogen bonds formed between the oxygen atom of Ala767 and the guanidium group of Arg776/Arg779 during the simulations of the wild-type and insertion mutant inactive EGFRs. (**C**) The distance between the main-chain oxygen atom of Ala767 and the Cζ side-chain atom of Arg776/Arg779 during the wild-type and mutant simulation.

In the wild-type inactive EGFR structure, a hydrogen bond between the guanidinium group of Arg776 (Arg779 in the insertion mutants) of the αC-β4 loop and the main-chain oxygen atom of Ala767 of the αC helix is regarded as autoinhibitory [62], anchoring the αC helix in the “αC-out” conformation. The structural models of the inactive state mutant EGFRs introduce a residue—*Ala770* in V769insASV EGFR and Asp770 in D770insNPG EGFR—near Arg779 that could potentially interfere with hydrogen bonding between the side chain of Arg779 and the main-chain oxygen atom of Ala767 (Figure 11A). Indeed, in the wild-type simulation the Ala767–Arg776 hydrogen bond was maintained for 53% of the simulation time, while the Arg779–Ala767 hydrogen bond in both insertion mutants was non-existent as it was recorded only for 1.33% (D770insNPG EGFR) and 0% (V769insASV EGFR) of the simulation time (Figure 11B). The distance between the main-chain oxygen atom of Ala767 and the Cζ side-chain atom of Arg776 (wild type)/Arg779 (mutant) also revealed a distant positioning of the two residues in the V769insASV (average distance of 8.4 ± 1.9 Å) and D770insNPG EGFR (5.4 ± 0.5 Å) simulations, as compared to wild-type EGFR (4.5 ± 1.0 Å) (Figure 11C). In the simulation of the EGFR D770insNPG inactive model structure, it was evident that the loop at the end of the αC helix and the placement of Asp770 in close proximity to Arg779 sterically prevents the optimal orientation of the Arg779 side chain for interaction with Ala767. Similarly, in the V769insASV inactive EGFR simulation, the inserted alanine (*Ala770*) acted as a wedge between Ala767 and Arg779 (Figure S6). These findings suggest that the insertion mutations might destabilize the inactive conformation of the kinase domain by preventing an interaction key to maintaining the inactive kinase state, which could then steer a transition towards the active kinase conformation.

## 4. Discussion

Non-synonymous somatic mutations of EGFR observed in cancer patients have been reported to alter the normal EGFR function by increasing kinase activity. One key category of EGFR mutation linked to NSCLC is the exon 20 insertion mutations, which include an array of small insertions that differ in the location of the insertion site and the number of inserted residues [29,30,31,32]. In comparison to the classical EGFR mutations, data on the insertion mutations are limited, especially when considering the structural aspects of the mutations. In this study, we aimed to uncover the structural implications of two frequently NSCLC associated EGFR exon 20 insertion mutations; D770insNPG and V769insASV that are located at the beginning of the αC-β4 loop and the end of the αC helix. By probing the dynamic motions of the active and inactive states of the wild-type and insertion mutant EGFRs, we aimed to dissect the local and global structural changes arising from the mutations and possibly pinpoint the mechanisms by which the mutations increase EGFR kinase activity. 

The simulations on the active state of wild-type and insertion mutant EGFRs revealed distinct differences on the state of key structural elements of the EGFR kinase domain, such as the αC helix, DFG motif and the Lys745–Glu762 salt bridge. The insertions were shown to stabilize the active state αC helix through formation of an additional turn of the α helix in the case of V769insASV, formation of a β-turn in the case of D770insNPG, and via other local interactions. The impact of the mutations on the αC helix can be attributed to the increased number of interactions at the site of the insertions that could help to firmly maintain the active state conformation of the αC helix. Furthermore, the positional and structural stability of the αC helix, imparted by the insertion mutations, led to the better conservation of the catalytically important Lys745–Glu762 salt bridge. 

The D770insNPG and V769insASV mutations also had an impact on the state of the DFG motif and R-spine. During the simulations of the insertion mutants, the active state orientation of the DFG motif and the arrangement of the R-spine residues were preserved. In contrast, with wild-type EGFR, Phe856 of the DFG motif attained an orientation similar to the one observed in crystal structures of the inactive EGFR kinase and Met766 on the αC helix was also displaced from the R-spine cluster of hydrophobic interactions. These findings can be credited to the flexibility of the αC helix recorded for the wild-type EGFR, which allows more room for Phe856 of the DFG motif to attain a different orientation especially as its hydrophobic partner, i.e., Met766, moves away along with the αC helix when transitioning towards the inactive kinase conformation. The analysis on the DFG motif was also a gentle reminder of the importance of performing independent simulations to sample a broader conformational space, as the change from the active towards the inactive state Phe856 orientation was sampled in one of the two simulations.

The inactive simulations did not pinpoint significant differences in the stability of the αC helix and the state of the Lys745–Glu762 interactions between the wild-type and insertion mutant EGFRs. However, in V769insASV EGFR, Phe856 of the DFG motif was observed to briefly visit a side-chain orientation similar to the active state. More importantly, the inactive state simulations revealed that the insertions could hinder the formation of an anchoring autoinhibitory interaction between Ala767 and Arg776/Arg779, which may help shift the equilibrium from the inactive EGFR conformation towards the active conformation. The Ala767–Arg776 interaction also takes place in the wild-type active EGFR simulation, however it is five-times less frequently observed than in the inactive conformation (10% versus 53% of the simulation time), which may suggest a pronounced role in the inactive EGFR state. In both the active and inactive state mutant EGFRs the Ala767–Arg779 interaction is nearly abolished due to the inserted residues, which might particularly affect the integrity of the inactive kinase domain conformation.

The majority of EGFR exon 20 insertion mutations exhibit reduced sensitivity to first- and second-generation TKIs, most notably to gefitinib and erlotinib [29,30]. On the other hand, the mutations have shown better sensitivity to osimertinib and poziotinib, with the latter exhibiting superior efficacy in in vitro and in vivo experiments [34,35,36]. In a few exon 20 insertion mutations, the replacement of the Asp770 residue with glycine has resulted in an enhanced efficacy towards the covalent inhibitor dacomitinib, highlighting the likely role of Asp770 in dacomitinib sensitivity [63]. The differential response of the insertion mutants to TKIs, in particular the resistance towards gefitinib and erlotinib, remains a topic for a thorough investigation. Our dynamics data on apo EGFR hint at an alteration near the ligand-binding pocket of the D770insNPG and V769insASV EGFRs, resulting from the change in the relative orientation of the N-lobe with respect to the C-lobe (Figure S7). In particular, the P-loop is seen closing on the binding pocket by approaching the A-loop of the C-lobe, which could have an impact on the access to the binding site and/or creating a steric hinderance to flexible ligands, such as gefitinib. Furthermore, the positional shift of the N-lobe structures, including the β3 and β4 strands, could affect ligand binding and interactions, consequently determining the sensitivity to TKIs. 

The findings described in this study for the D770insNPG and V769insASV EGFR exon 20 insertion mutations have suggested an overall similar structural impact, with concerted inward movements of key features of the kinase structure—most of the N-lobe structural elements, such as the P-loop, β3-αC loop, β4-β5 loop, and the β3 and β4 strands as well as the state of the DFG motif of the C-lobe—that are important for maintaining an activated kinase. These observations might not be entirely extrapolated to all other EGFR exon 20 insertion mutations, which occur at slightly different locations relative to the D770insNPG and V769insASV mutations. Hence, a detailed structural characterization on the spectrum of exon 20 insertion mutations could provide additional mechanistic insights on the mutations that play a significant role in the pathogenesis of NSCLC and the role of these mutations in altered TKI sensitivity. 

## 5. Conclusions

This study has examined the dynamic structural changes exerted by two prominent activating EGFR exon 20 insertion mutations; D770insNPG and V769insASV. The molecular dynamics study has also uncovered the mechanisms by which the mutations alter EGFR kinase activity. The two mutations, located at the C-terminus of the EGFR αC helix and N-terminus of the αC-β4 loop, affected the active state of the protein by imparting structural and positional stability to the αC helix, by maintaining the Lys745–Glu762 salt bridge and the conformation of the DFG motif. These features were compromised in the wild-type EGFR simulation. In the inactive state EGFR, the insertion mutations resulted in the breakage of a key autoinhibitory interaction between Ala767 and Arg776, which could promote an equilibrium shift from the inactive towards the active EGFR conformation. In conclusion, our study suggests that the D770insNPG and V769insASV insertion mutations increase EGFR kinase activity possibly by affecting both the active and inactive states of the EGFR kinase conformation.

## Data Availability

Data are contained within the article or supplementary material.

## Supplementary Materials

The following material is available online at https://www.mdpi.com/2072-6694/13/5/1120/s1, Figure S1: Correlation between the motions of residues of the active wild-type and insertion mutant EGFRs, Figure S2: Conformational flexibility of the β3-αC loop and structural integrity of the αC helix, Figure S3: The conformation of and distance between V769 and L828/L831 during the active state wild-type and insertion mutant EGFRs, Figure S4: Secondary structure representation of the αC helix residues during the inactive wild-type, D770insNPG and V769insASV EGFR simulations, Figure S5: Superimposed sampled conformations from the inactive wild-type, D770insNPG and V769insASV EGFR simulations, Figure S6: Sampled conformations at 100, 300 and 500 ns of the inactive wild-type and insertion mutant EGFR simulations showing the A767-R776/R779 interaction, Figure S7: Superimposed conformations sampled at 85, 300 and 500 ns of the simulations for the apo wild-type and insertion mutant EGFRs, File S1: Active wild-type EGFR at 85 ns, File S2: Active wild-type EGFR at 300 ns, File S3: Active wild-type EGFR at 500 ns, File S4: Active D770insNPG EGFR at 85 ns, File S5: Active D770insNPG EGFR at 300 ns, File S6: Active D770insNPG EGFR at 500 ns, File S7: Active V769insASV EGFR at 85 ns, File S8: Active V769insASV EGFR at 300 ns, File S9: Active V769insASV EGFR at 500 ns, File S10: Inactive wild-type EGFR at 100 ns, File S11: Inactive wild-type EGFR at 300 ns, File S12: Inactive wild-type EGFR at 500 ns, File S13: Inactive D770insNPG EGFR at 100 ns, File S14: Inactive D770insNPG EGFR at 300 ns, File S15: Inactive D770insNPG EGFR at 500 ns, File S16: Inactive V769insASV EGFR at 100 ns, File S17: Inactive V769insASV EGFR at 300 ns, File S18: Inactive V769insASV EGFR at 500 ns.
