# Supplementary material for: Structural Basis for the Functional Changes by EGFR Exon 20 Insertion Mutations
Source: Cancers (Basel). 2021 Mar 5;13(5):1120. doi: 10.3390/cancers13051120 (PMC7961794; doi:10.3390/cancers13051120)
Supplement: Supplementary file 1 [file cancers-13-01120-s001.zip › cancers-1093462-supply/cancers-1093462-supply.docx]

Supplementary Materials

Structural Basis for the Functional Changes by EGFR Exon 20 Insertion Mutations

Mahlet Z. Tamirat, Kari J. Kurppa, Klaus Elenius and Mark S. Johnson


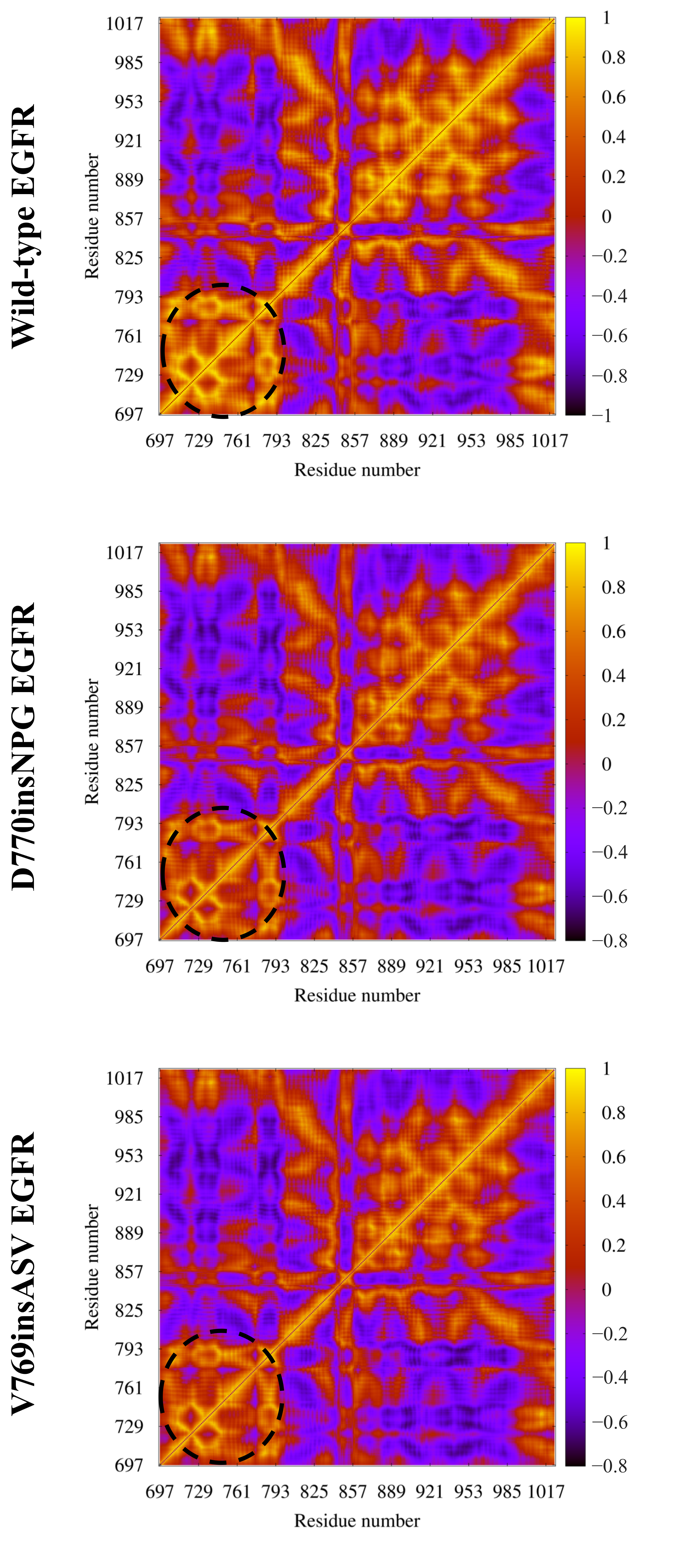


**Figure S1.** Correlation between the motions of residues of the active wild-type and insertion mutant EGFRs. Motions from correlated to non-correlated to anti-correlated are colored as a gradient from yellow to brown to black. The amino acids of the kinase domain N-lobe (encircled) show highly correlated motions during the wild-type, D770insNPG and V769insASV EGFR simulations.


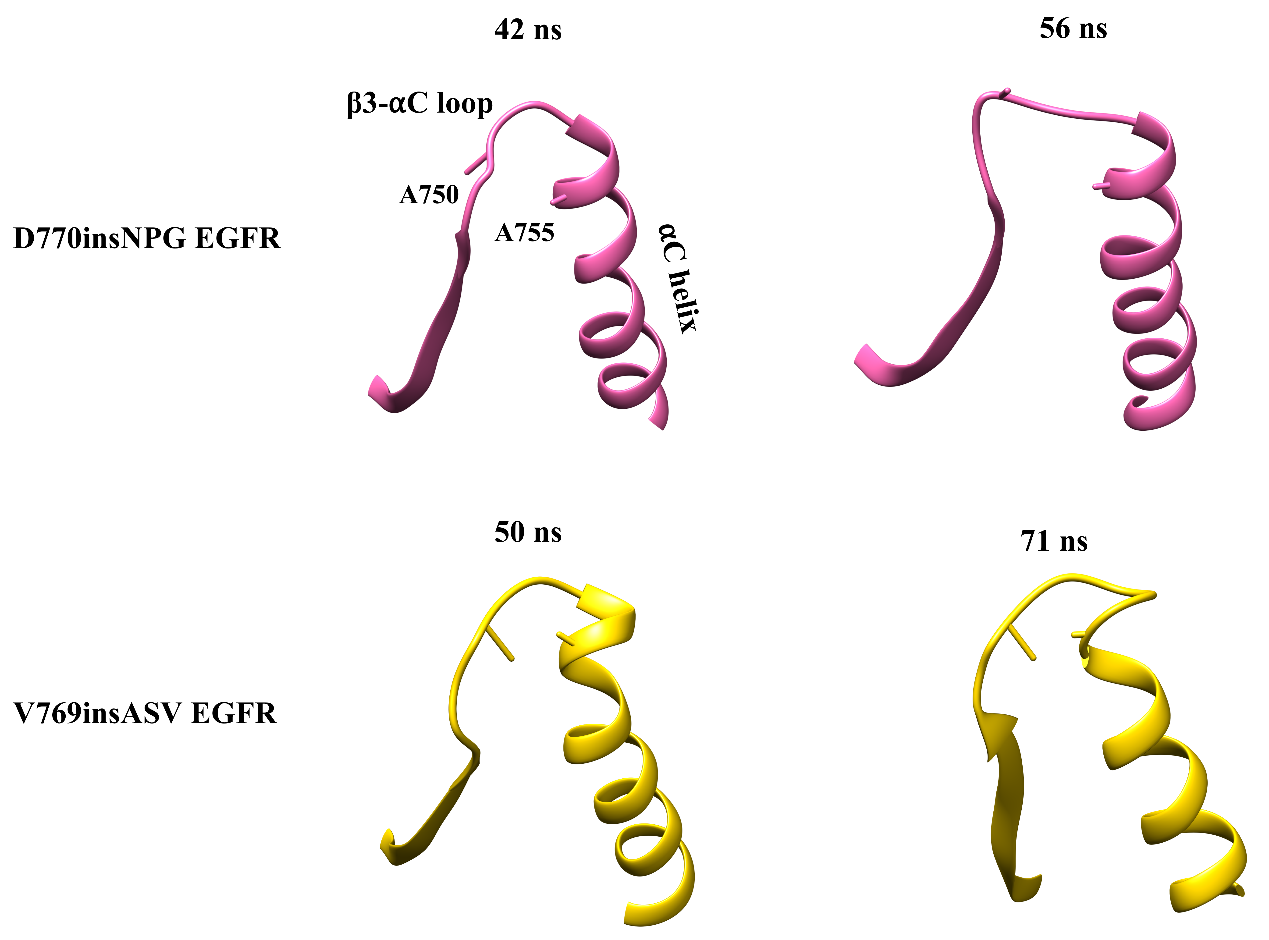


**Figure S2.** Conformational flexibility of the β3-ɑC loop and structural integrity of the ɑC helix. Randomly sampled conformations during the active A) D770insNPG and B) V769insASV EGFR simulations. In D770insNPG EGFR, A750 of the β3-ac loop and A755 of the ɑC helix are distantly placed allowing the β3-ɑC loop to attain different conformations and the N-terminal part of the ɑC helix to conserve the helical structure. In contrast, in V769insASV EGFR there is a close interaction between the two alanine residues, which makes the β3-ɑC loop attain a similar conformation during the simulations, hence a lower flexibility. The ɑC helix on the other hand loses integrity of the first turn of the helix as a result of maintaining the A750-A755 interaction.


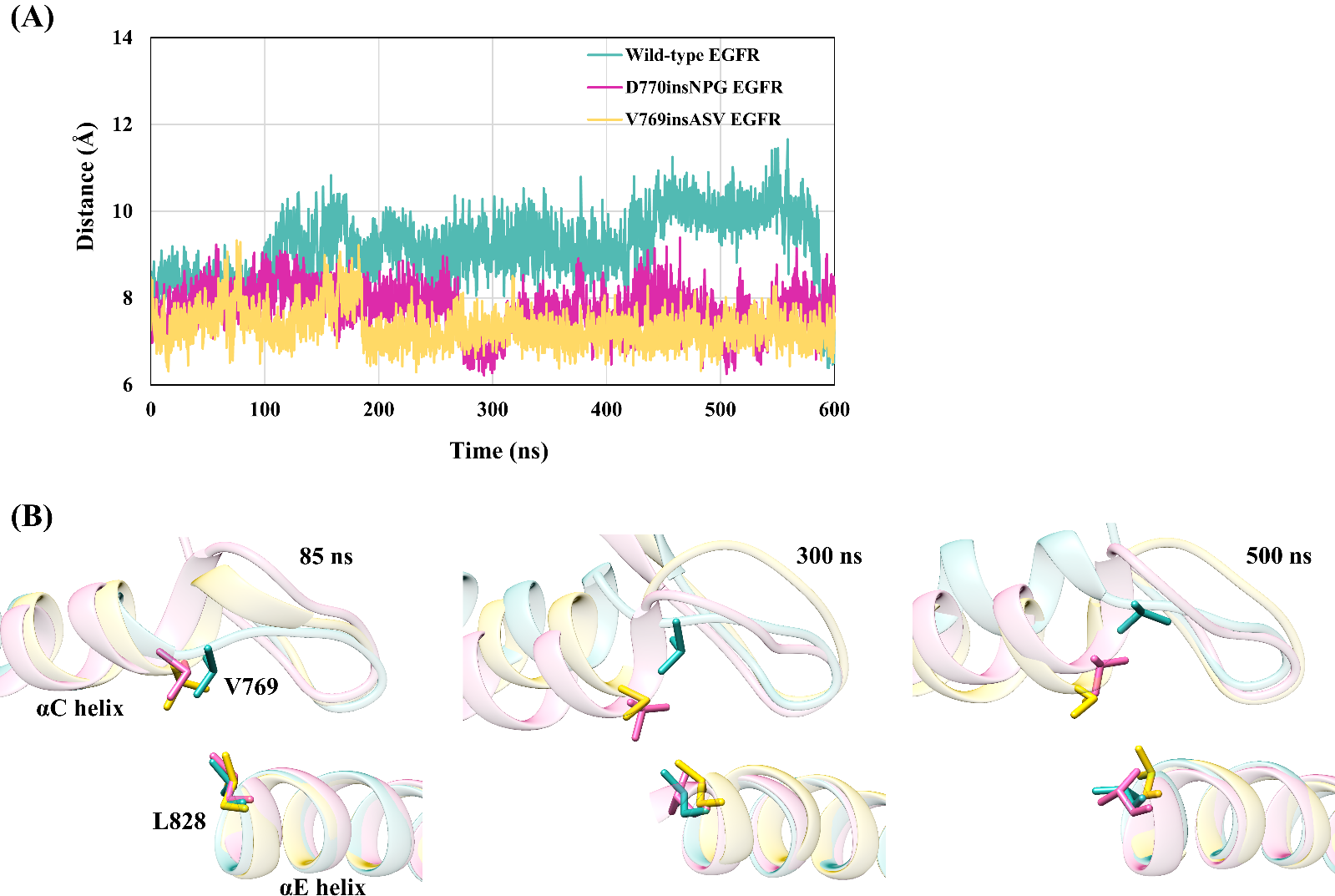


**Figure S3.** Conformational changes affect the interaction between V769 and L828 (wild type)/L831 (mutant) during the active state wild-type and insertion mutant EGFRs. (**A**) Distance between the Cɑ atoms of V768 and L828/L831 during the wild-type, D770insNPG and V769insASV EGFR simulations. (**B**) Sampled conformations at 85, 300 and 500 ns of the active state simulations showing the orientations of V769 and L828/L831 residues.


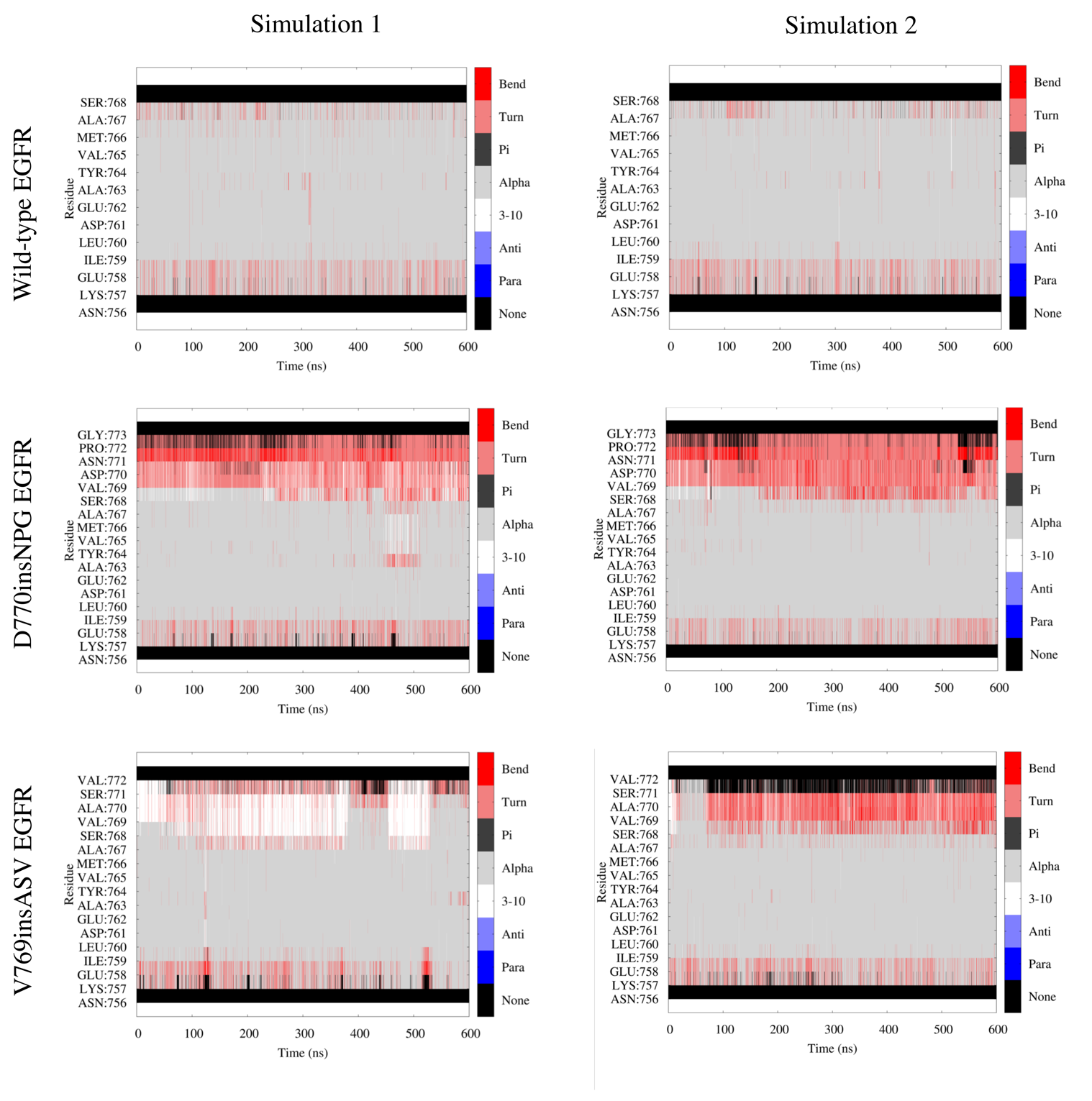


**Figure S4.** Secondary structure representation of the ɑC helix residues during the inactive wild-type, D770insNPG and V769insASV EGFR simulations. The structural integrity of the ɑC helix is largely comparable among the three EGFRs, with the central part conserving the helical structure and the N-terminal end exhibiting turns and bends. At the C-terminal end of the ɑC helix of the insertion mutants the inserted resides included, which largely adopt turns and bends. Multiple frames also show a 3–10 helix structure at this region, particularly for the V769insASV EGFR residues.


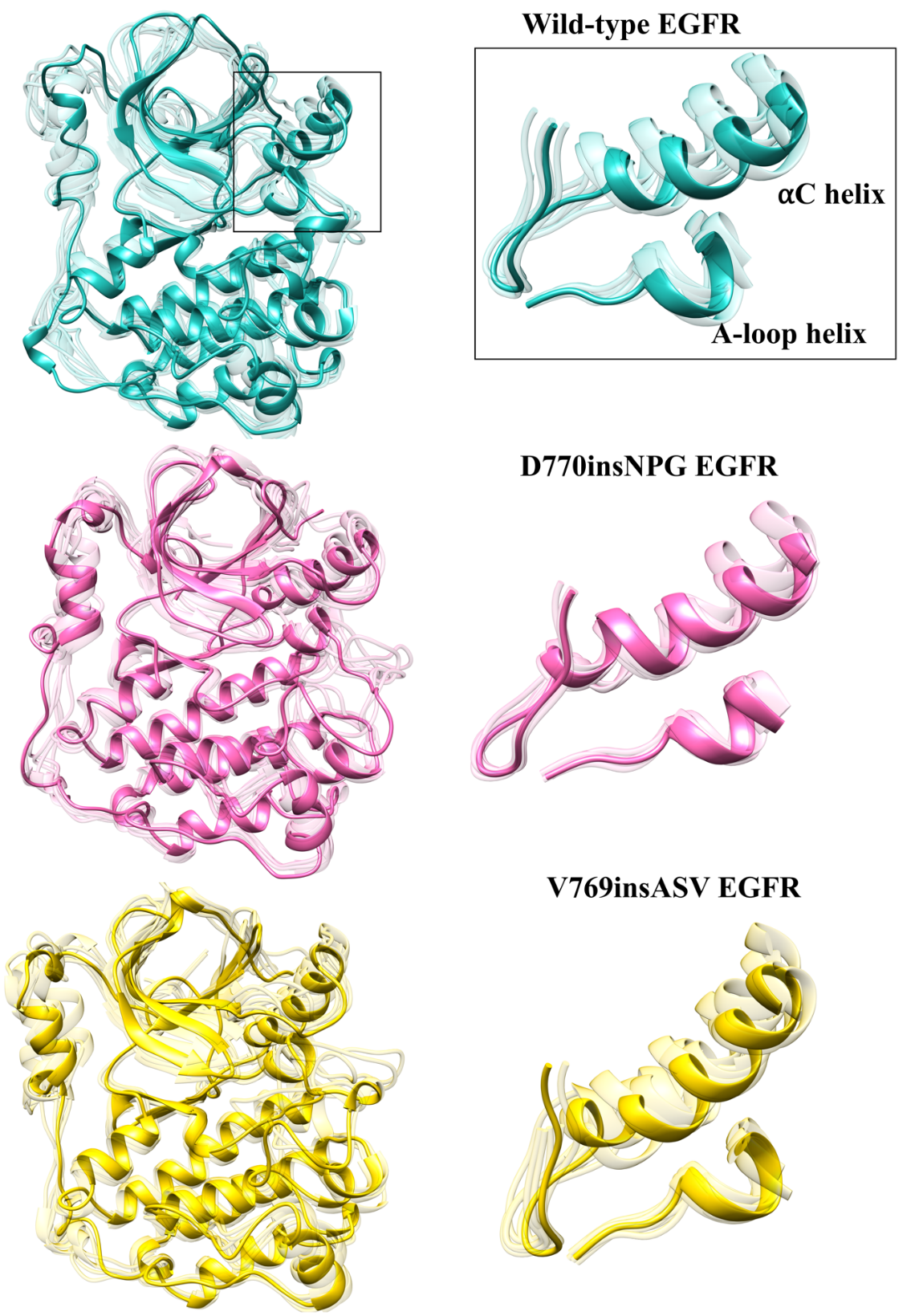


**Figure S5.** Superimposed sampled conformations from the inactive wild-type, D770insNPG and V769insASV EGFR simulations. The median sampled structure (at 300 ns) is shown is solid color, whereas the rest are faded (0, 100, 200, 400, 500 and 600 ns). In both the wild-type and insertion mutant EGFRs the inactive conformation is maintained, with the ɑC helix in the “ɑC-out” state and an intact A-loop helix.


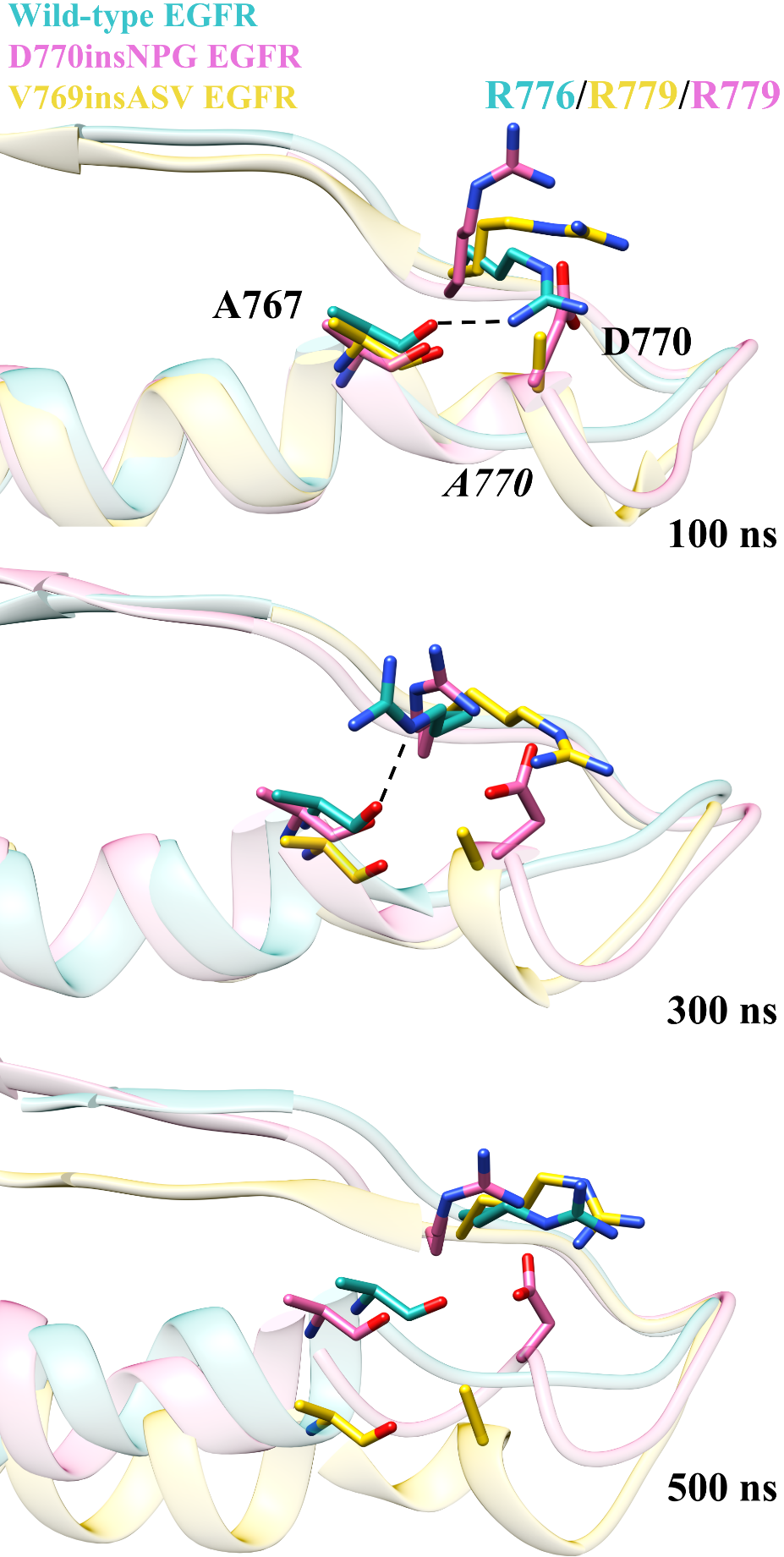


**Figure S6.** Sampled conformations at 100, 300 and 500 ns of the inactive wild-type and insertion mutant EGFR simulations showing the A767-R776/R779 interaction. Residues A770 in V769insASV EGFR and D770 in D770insNPG EGFR hinder the formation of the hydrogen bond between A767 and R779. In the wild-type EGFR simulation the A767-R776 interaction (dotted line) is frequently formed.


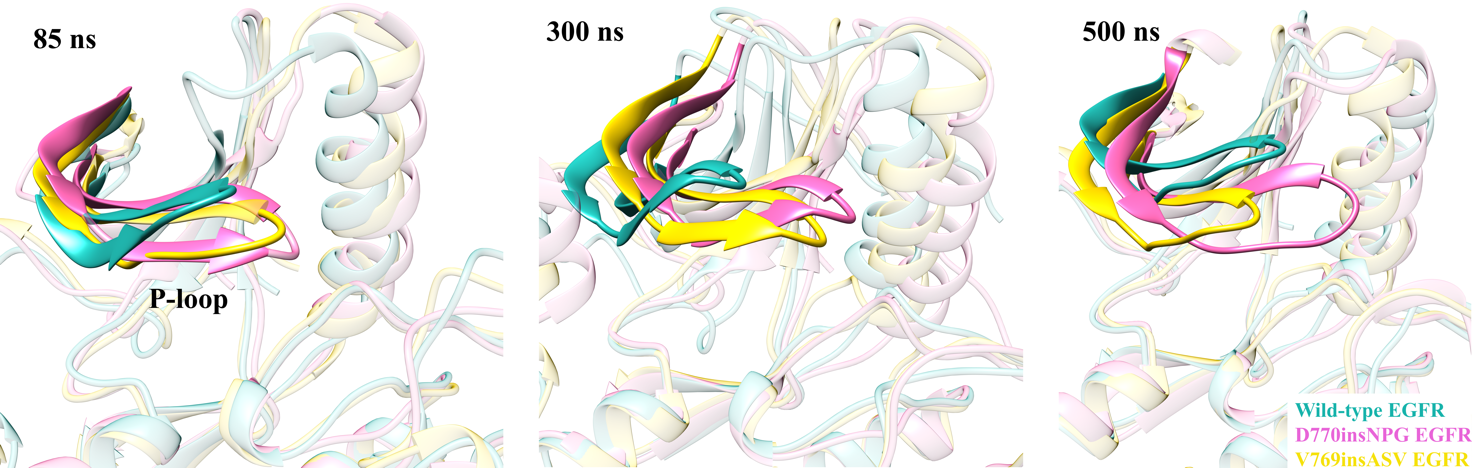


**Figure S7.** Superimposed conformations sampled at 85, 300 and 500 ns of the simulations for the apo active wild-type and insertion mutant EGFRs. The P-loop of the kinase domain is observed to adopt a different conformation (a positional shift) in the D770isNPG and V769insASV mutants in comparison to wild-type EGFR. The P-loop is seen in closer proximity to the A-loop in the mutants than in wild-type EGFR. This is likely linked to the overall shift of the N-lobe relative to the C-lobe in the mutant EGFRs.

| 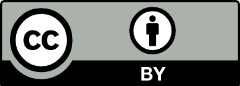 | © 2021 by the authors. Licensee MDPI, Basel, Switzerland. This article is an open access article distributed under the terms and conditions of the Creative Commons Attribution (CC BY) license (http://creativecommons.org/licenses/by/4.0/). |
| --- | --- |
